# The Role of Reverse Osmosis as an Essential Desalination Technology in Addressing Spain’s Freshwater Deficits

**DOI:** 10.3390/membranes16040113

**Published:** 2026-03-24

**Authors:** Antonio Casañas Gonzalez, Veronica García Molina, Federico Antonio Leon Zerpa, Alejandro Ramos Martin

**Affiliations:** 1 Dow Water and Process Solutions, Dow Ibérica S.L., 28042 Madrid, Spain; tonicasanas@hotmail.com (A.C.G.); veronica.garciamolina@dupont.com (V.G.M.); 2 Industrial Engineering School, University of Las Palmas de Gran Canaria, 35001 Las Palmas de Gran Canaria, Spain; alejandro.ramos@ulpgc.es

**Keywords:** membranes, reverse osmosis, desalination, water reuse

## Abstract

Water is increasingly acknowledged as a limited and strategically critical resource, particularly in regions where hydrological imbalances are structurally persistent. Across Europe, countries such as Spain, Turkey, Italy, and Greece face recurrent water scarcity driven by precipitation regimes characterized by low annual rainfall, pronounced temporal variability, and marked spatial heterogeneity. In response to rising water demand associated with tourism, agricultural intensification, and sustained demographic pressures, Spain has implemented a series of national water-management strategies over the past two decades. Notably, the National Hydrological Plan, enacted in July 2005, introduced more than one hundred immediate actions focused on modernizing hydraulic infrastructure and reinforcing the country’s desalination capacity. Furthermore, the Royal Decree issued in December 2007 established a comprehensive regulatory framework to promote and standardize water reuse practices nationwide. Within this context, reverse osmosis has emerged as a central technology for the desalination of seawater and brackish water, as well as for advanced water-reclamation applications. This work presents a consolidated examination of Spain’s water-resource management framework, drawing on historical material and recent advances to outline the current context of desalination and water reuse. It presents operational performance data from several full-scale reverse osmosis facilities, and reviews recent technological developments in the field, including newly engineered membrane modules, innovative system architectures, and the latest generation of large-diameter RO elements. Together, these advancements illustrate the evolving role of membrane-based desalination and water reuse in supporting water security in semi-arid regions.

## 1. Water Resources in Spain

This study aims to provide an integrated and up-to-date assessment of the role of reverse osmosis (RO) within Spain’s evolving water-resource management strategies. In regions facing persistent hydrological imbalance, the increasing reliance on membrane-based desalination and water-reuse technologies has become essential for ensuring long-term water security. The objective of this work is therefore threefold: (i) to contextualize Spain’s current water-scarcity challenges and the policy frameworks that have shaped national responses; (ii) to present operational performance data from representative full-scale RO facilities operating across the country; and (iii) to review recent technological advancements in desalination, including innovations in membrane module design, system architectures, and large-diameter RO elements. By combining policy analysis, operational evidence, and technological review, this paper delineates the scope and potential of RO technologies as a strategic cornerstone for sustainable water supply in semi-arid environments.

Spain has been suffering from a tendency of increasing water scarcity due to the rise in population and tourism (especially in the coastal areas), augment of areas dedicated to agricultural activities, diminishing rainfalls, and a water resource policy not fully optimized. As described elsewhere [1], the “water stress index” is the ratio between the average amount of withdrawal and the amount of long term available fresh water. Spain has been reported to have a water stress index of around 30%, being 40% the situation of acute water scarcity and 10% a sustainable scenario. Countries such as Belgium, Malta, and Cyprus have water stress indexes above 40%, whereas the United Kingdom, Netherlands, Slovenia, Austria, Ireland, and Sweden present values below 10%.

**Figure 1:**
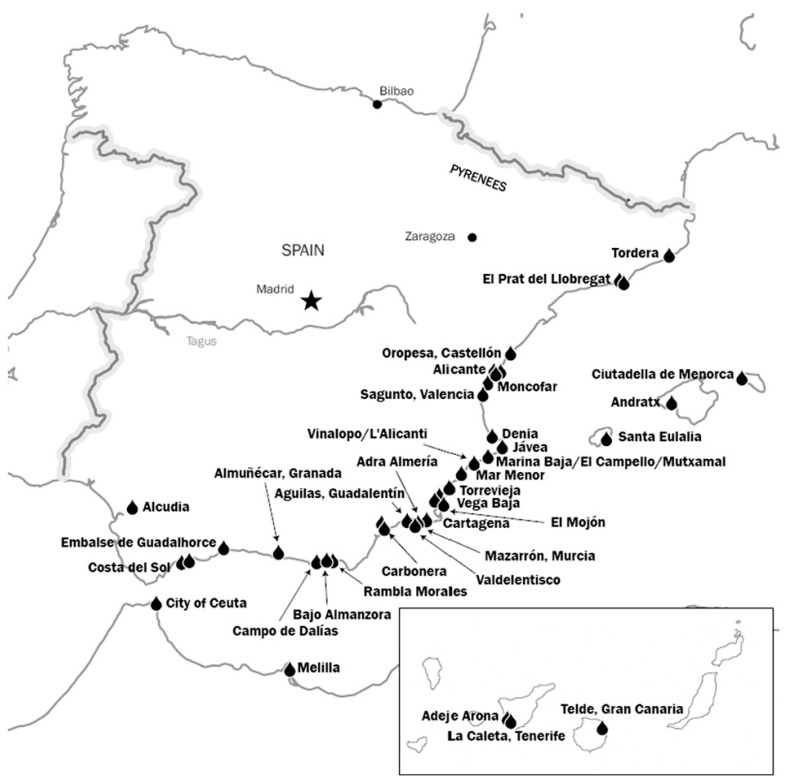
Main seawater Reverse Osmosis desalination plants in operation or under construction in Spain.

In order to remediate the poor hydrologic situation of the country, especially of the Mediterranean area, a first attempt was taken by the Government back in 2001 with the “Plan Hidrológico Nacional” (National Hydrologic Plan). Among others, one of the main strategies included in the Plan was the transfer of water from River Ebro to different areas of the Mediterranean coast [2]. In 2005, the current Socialist Government claimed that the initial Hydrologic Plan had underestimated the ecological impact on protected areas of River Ebro and that the final costs of the project and consequently, the final cost of the transferred water were higher than the initial expectations (0.39 vs. 0.91 EUR/m^3^ [3]). The initial Plan was consequently modified and a new strategy mainly based on seawater and brackish water desalination, water reuse and on the improvement, modernization, and expansion of existing facilities, conductions, and water supply systems was included first in a Royal Decree and later in an Organic Law [4]. In total, more than 100 points of action were identified in the modified National Hydrologic Plan under the WATER program. Among these actions, the construction of large seawater reverse osmosis desalination plants such as Campo de Dalías with a capacity of 100,000 m^3^/day, Marina Baja with 50,000 m^3^/day or Costa del Sol with 60,000 m^3^/day can be distinguished. Figure 1 shows some of the most important seawater desalination plants already in operation or under construction and in Table 1, the planned seawater desalination plants and water reuse installation included in the Organic Law of June 2005 as priority actions are included.

Some other actions are included in the WATER program point at water reuse applications such as the projects in Almería, Málaga, Murcia, and el Mar Menor. More specifically, the Government’s WATER program sets a target of providing an additional 137 million m^3^/y from reuse on a national scale. As an example, two tertiary wastewater treatment plants, San Javier with a capacity of 30,000 m^3^/day and Los Alcázares (22,500 m^3^/day) have been recently completed and Hellín, with a capacity of 12,500 m^3^/day has just gone out to tender [5]. At December of 2007 it was approved a Royal Decree for Water Reuse [6], further treatment of the WWTP tertiary effluents is going to be needed in order to achieve the new challenging quality requirements to make possible the reutilization of the treated water. In fact, the new Royal Decree defines specific minimum water quality requirements depending on the water reuse application. Even though Reverse Osmosis has been applied in many water reuse installations, it is currently one of the most promising technologies in order to accomplish this stricter Legislation.

Water Reuse, in general, is not equally practiced in the different provinces of the Spanish territory. As an example, in the area of the Segura river basin, 200 million m^3^/y of wastewater is treated annually and around 95% of it is currently reused. Of the total amount of water reused, 50% is used for irrigation and the rest is channeled into the Segura River, which serves again for irrigation purposes further downstream. It is especially important to emphasize the water reuse tradition of the area and to remark that only part the total capacity has been created as a result of the WATER program. On the other hand, areas such as Cataluña, with a lower agricultural activity, reused directly only 5% of its wastewater in 2006 [5].

**Figure 2:**
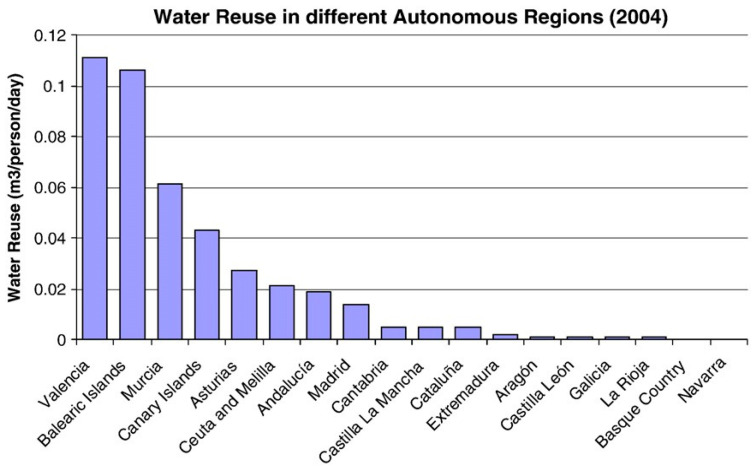
Geographical distribution of water reuse in m^3^/person/day.

In Figure 2 the amount of water reused in 2004 in the different Autonomous Regions of Spain is shown. From this picture it can be concluded that the areas suffering from a more acute water stress situation, i.e., Júcar and Segura Rivers basins and the Balearic and Canary Islands, are the regions where Water Reuse has been developed the most. In order to promote water reuse within Cataluña and to improve the old situation of the natural water resources, the Autonomous Government approved in February of 2008 the construction of 25 urban wastewater treatment plants. The project included 20 installations in the province of Lleida and 5 in Tarragona.

Undoubtedly, Reverse Osmosis is playing a fundamental role to ensure sufficient and reliable water supply for all the different purposes (irrigation, potable, process water, etc.). Together with the growth of the technology implementation, certain unmet market needs start to acquire more and more importance. In this sense, innovations in Reverse Osmosis are currently guided by different strategies in order to achieve a reduction in the Capital and Operating expenses, i.e., CAPEX and OPEX of the plants.

## 2. Seawater Reverse Osmosis: New Modules and Configurations

In the 90s, the price of RO desalinated water was estimated to be 1.50 USD/m^3^ whereas nowadays, certain plants are producing water at a total cost of around 0.5 USD/m^3^. A high percentage (between 45 and 55%) of the total cost of the water is electricity consumption. It is important to emphasize that among the total power consumption, between 80 and 90% corresponds to the high-pressure pump. It is thus reasonable to focus the invested efforts in decreasing the final price of water in the reduction in the needed feed pressure [3,4].

**Figure 3:**
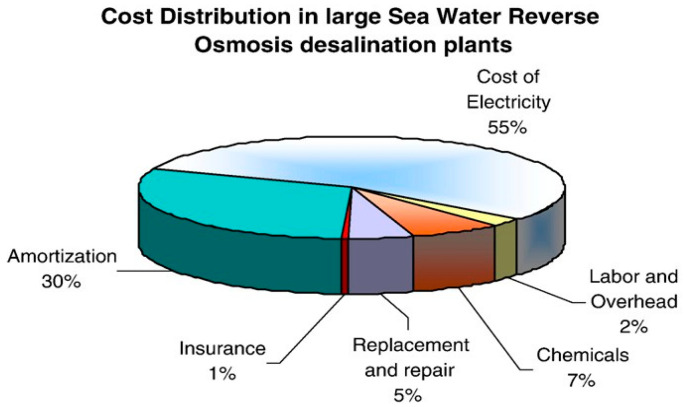
Cost distribution in large Seawater Reverse Osmosis desalination plants.

An evaluation of the contribution to the final cost of water of the operating and capital expenses is shown in Figure 3. For this evaluation, a SWRO (Seawater Reverse Osmosis) plant (currently under tender) with a total capacity of 150,000 m^3^/day, 37,000 ppm feed TDS, temperature range 14–25 °C, and strict permeate quality of Bromide below 0.1 ppm was chosen. The results shown in Figure 3 are the average values of more than 20 different designs evaluated for this particular plant. From these results, it can be concluded that the operating costs including cost of electricity, insurance, replacements and repairs, labor, overhead, and chemical consumption stand for 70% of the final cost of water [3,5,6,7,8].

Efforts have been made over the last years in order to reduce the high energy consumption of SWRO plants to make desalination more affordable. As a comparison, in the 80s, the energy recovery devices had an average efficiency of 75%, whereas the latest models can recover about 96% of the energy from the waste stream [3,8].

### 2.1. Recently Developed Seawater Reverse Osmosis Modules

From the point of view of Reverse Osmosis membrane manufactures, the goal is to produce modules with the highest productivity and the highest rejection. In order to reduce the feed pressure demand, Dow Water Solutions recently launched the ultra-low energy seawater modules SW30ULE-400i, with a production of 11,000 gpd and 99.70% salt rejection under standard test conditions (32,000 ppm TDS, pH 8, 800 psi, 8% recovery, and 25 °C). These high productivity modules represent important advantages in terms of energy savings in two different scenarios:

□ Low temperature desalination. Under these conditions and due to the low temperature, the salt passage through the membrane is relatively low and consequently, the required quality is easily achieved. However, and again due to the low temperature, the feed pressure required is usually considerably high. Modules with a higher production can afford a reduction in the feed water pressure without compromising the final quality of the water.
□ Seawater desalination plants with two passes. In these installations, the required quality of the permeate is relatively high and a second pass is needed in order to further treat the permeate obtained in the first pass. With the installation of high productivity modules in the first pass, the feed pressure and thus, the total energy consumption of the plant, can be significantly reduced. Due to the lower rejection of these modules compared to standard seawater modules, the size of the second pass may increase; however, the benefit obtained from the reduction in the OPEX compensates the increase in the CAPEX.

Regarding the quality of the permeate produced, Boron has played an important role over the last years. The limit for Boron in drinking water was fixed by the EU at 1 ppm while the WHO recommended a value of 0.5 ppm (although this limit is expected to be increased in the near future). Historically, in the Mediterranean countries, a second pass was needed in SWRO plants in order to ensure a Boron content in the permeate within the limits. Nowadays and thanks to the recently developed FILMTEC^TM^ high Boron rejection modules, SW30XHR-400i, it is possible to achieve Boron limits below 1 ppm in single pass configurations with the consequent savings in OPEX and CAPEX. This module has the highest proven Boron rejection in the seawater market [8,9,10].

The first 500 FILMTEC^TM^ SW30XHR-400i modules were installed in January 2008 in a SWRO plant in Murcia. This plant (single pass) takes the feed water from a well with a conductivity of 48,100 µS/cm and a Boron content of 4.84 ppm. In recent modifications of the plant, the old Pressure Exchange device was replaced by an Energy Recovery Unit. Because of this, the feed salinity and Boron content of the feed is estimated to increase up to 3%. In Table 2, the operating conditions of this plant are summarized. This Seawater plant constitutes an excellent reference due to the achievement of such low Boron content in the permeate within one pass [11,12,13,14].

### 2.2. Internally Staged Designs

The concept of Internally Staged Designs for Seawater Reverse Osmosis plants (Dow Patent pending; application number: PCT/US2005/006224) was already introduced some time ago as an economically attractive alternative to decrease the capital and the operating costs of Seawater Desalination [3,8,9]. The principle of Internally Staged Designs consists of installing elements with different productions along the pressure vessels. Modules with a lower production are placed in the first positions to ensure a low flux and thus, minimizing fouling risk and elements with higher production in the subsequent positions of the vessel. The effect of such a design in a pressure vessel is similar to a two-stage design with a booster in the 2nd stage or a permeate back pressure in the 1st stage. The result is a more compensated flow production inside the vessel, or in other words, the first elements are less prone to fouling and concentration polarization problems while the rear elements have a higher contribution to the total permeate production. Obviously, reducing the productivity of the first several elements in series, while keeping the same overall vessel productivity, suggests some different ways of operating the vessel. A designer could try to save in operating costs by decreasing the operational pressure. Another route could be using the improved hydraulic situation and increase the vessel productivity with a higher flux (flow per membrane area) and/or with a higher recovery (higher throughput). In both cases the limits for flow and recovery which are set by the engineering guidelines have to be respected [3,8,15,16].

In order to provide the reader with a better understanding of the benefits of Internally Staged Designs, a comparison between three extreme configurations has been made. With the aim of having a readily comparable system, the operation parameters have been kept constant for all the simulations. The three configurations are described below:

□ Standard Design with SW30HRLE-400i elements. This example constitutes the standard design for SWRO desalination plants, where SW30HRLE-400i elements with a production of 7500 gpd and 99.75% rejection are used in the first pass.
□ Standard Design with high productivity modules SW30ULE-400i with a standard production of 11,000 gpd and 99.7% salt rejection.
□ Internally Staged Design with one module SW30HRLE-400i in the first position, one module SW30XLE-400i and finally, four elements SW30ULE-400i were used in the rear positions. It is important to notice that the specified flow of the elements installed in this pressure vessel increases from feed to concentrate side.

**Figure 4:**
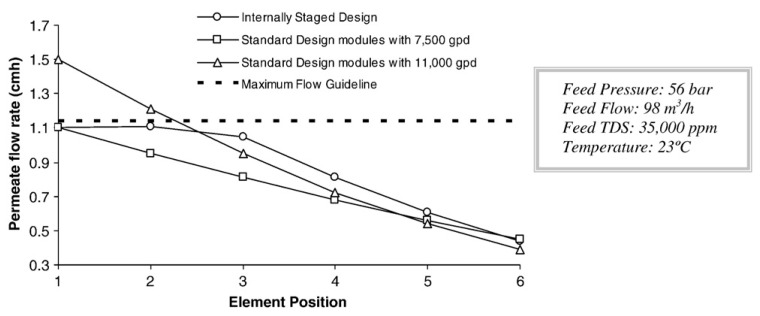
Comparison of flow distribution along pressure vessels between Internally Staged Design and two conventional configurations.

The permeate flow produced by the individual modules in each one of the three configurations is illustrated in Figure 4. As shown in the plot, the two first modules of the standard design with high productivity modules (SW30ULE-400i) operate at a flow higher than the recommendation. This might result in a prompt fouling of these elements with the consequent increase in the feed pressure demand and a frequent cleaning requirement. This problem is not observed with the standard design with SW30HRLE-400i modules and with the ISD configuration. However, for the same feed pressure demand, a higher amount of permeate flow is obtained when the ISD concept is applied [17,18,19].

**Figure 5:**
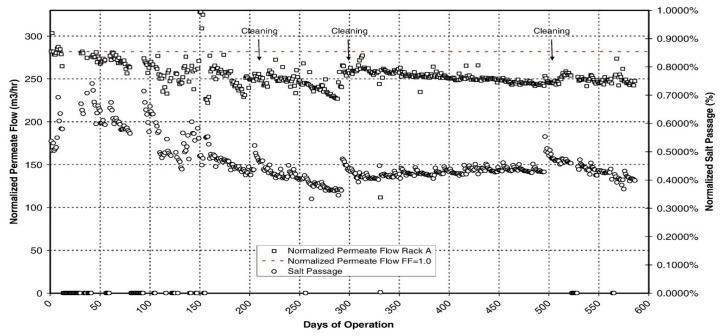
Normalized permeate flow and salt passage. 65,000 m^3^/day SWRO plant.

In Figure 5 the performance in terms of normalized Flow and Salt Rejection of one of the racks of a Seawater Reverse Osmosis Desalination plant in the Mediterranean region is shown. The plant, with a capacity of 65,000 m^3^/day has already been in operation for more than one year. It has an open intake and before the reverse osmosis, water is pre-treated with chlorination, coagulation + flocculation, pH regulation, sand filtration, dispersant addition, sodium bisulphate, and cartridge filters. The Reverse Osmosis racks were designed with an Internally Staged Design using two modules with a lower production and higher rejection in the first positions and modules with higher production and standard rejection in the last positions of the pressure vessels. During these first months of operation, both flow and rejection have been within the expectations and only three cleanings have been applied. The plant was started with a permeate production of around 280 m^3^/h reaching a final stabilized flow of 250 m^3^/h, meaning a fouling factor of around 0.9 after almost two years of operation. On the other hand, the initial salt passage was around 0.6% and once stabilized, it reached a value close to 0.4%. Punctual increases in the salt passage were observed right after each cleaning, but the stabilized value was attained again after few days of operation [20,21].

Another Spanish seawater plant using Internally Staged Design, with a total capacity of 70,000 m^3^/day was recently started. This plant uses a configuration of 6 + 1, i.e., six modules SW30HRLE-400 and one SW30XLE-400i. So far, four of the seven racks have been in operation, giving optimal results in terms of rejection.

Spain’s hydroclimate remains governed by strong inter-annual variability, recurrent multi-year droughts, and pronounced spatial heterogeneity between humid Atlantic basins and semi-arid Mediterranean catchments. Contemporary European assessments confirm that seasonal water scarcity persists across large parts of Southern Europe; the Water Exploitation Index Plus (WEI+) indicates stress conditions (WEI+ > 20%) in multiple Spanish river subunits during at least one quarter almost every year, with severe stress (WEI+ ≥ 40%) episodically attained in Mediterranean basins under combined agricultural, urban, and tourism pressures. These pressures are compounded by structural demand: agriculture accounts for the majority of abstractions and remains highly sensitive to heatwaves and precipitation deficits; macro-level analyses in 2025 characterized Spain’s situation as an “increasing water emergency,” calling for a sustained step-up in investment and demand-side efficiency [22].

Spain has completed a major regulatory update to harmonize reuse with the EU Regulation on minimum requirements for water reuse [6], which has applied across Member States since June 2023 and is complemented by technical risk-management specifications adopted in 2024. In October 2024, Spain enacted Royal Decree 1085/2024, replacing the RD 1620/2007 and establishing an authorization-based regime with risk-management plans, quality standards by use, monitoring and conformity criteria, and measures to promote reuse—particularly in the urban cycle. The Royal Decree explicitly derogated the RD 1620/2007 and operationalizes the EU regulation domestically; legal and policy notes emphasize transitional arrangements and expanded roles for producers, suppliers, and end-users of regenerated water. MITECO guidance released in 2025 supports the development of reuse risk-management plans and municipal promotion programs, anchoring reuse as a mainstream instrument of integrated water management.

Spain remains the EU’s leading adopter of membrane desalination. Sector snapshots in 2024–2026 report approximately 5.0 million m^3^/d of installed desalinated-water production capacity nationwide (seawater and brackish), with ~54 large SWRO plants (>10,000 m^3^/d each) concentrated along the Mediterranean arc and island systems; large facilities constitute ~77% of the total capacity. Press coverage and industry briefs converge on ~700–770 operational desalination plants country-wide (counting medium and small units), with roughly one hundred high-capacity installations. In response to the 2023–2024 Catalonian drought, emergency and structural programs are expanding SWRO capacity (e.g., Tordera II) and deploying modular units to hard-hit municipalities; Barcelona’s El Prat SWRO covered about one-third of metropolitan demand at the drought peak.

On reuse, Spain leads Western Europe and is systematically upgrading to meet EU 2020/741 classes and national provisions under RD 1085/2024. MITECO’s 2025 framework underscores the obligation to prepare urban reuse promotion plans and risk-management plans; sector narratives anticipate growth toward ~1000 hm^3^/y reclaimed water as investment programs mature. Policy analyses (2025) further recommend integrating non-conventional water (NCW) within basin planning and addressing adoption barriers (cost, governance fragmentation, social acceptance) [3,8,20,21,22,23].

Spain’s modern SWRO plants embody contemporary pretreatment schemes tailored to local seawater quality: dissolved air flotation (DAF) + dual-media filtration (DMF) trains, or pressurized ultrafiltration (UF) where variable intakes, footprint limits, or lower life-cycle cost justify membranes [3,8]. Full-scale monitoring shows that two-stage DMF effectively controls particulate fouling (SDI_15_, MFI_0.45_), while DAF + DMF improves removal of biopolymers and bacterial growth potential (BGP), correlating with lower biofouling risk in RO stages. Industry experience also documents cases in Spain adopting PVDF UF as pressurized pretreatment to stabilize RO feed quality (e.g., Maspalomas-I, Gran Canaria), with demonstrated low SDI filtrate and high recovery under variable open-intake conditions—an approach particularly relevant for insular systems with tourism peaks. Contemporary international design papers [23] benchmark pretreatment choices (UF vs. media filtration) using carbon footprint, brine/sludge disposal, treated-water quality, and total expenditure (TOTEX) as criteria—frameworks that Spanish EPCs are increasingly applying in expansions and retrofits [16].

On energy performance, current Spanish SWRO designs leverage isobaric energy-recovery devices and high-permeability membranes, often pursuing PV integration or PPAs to mitigate energy costs and decarbonize supply—an area highlighted in national drought responses and investment programs since 2024–2025 [3,8]. Global market intelligence indicates robust growth in RO and reuse technologies, with Spain positioned as Europe’s largest national market for desalination equipment by 2030, driven by structural scarcity and modernization needs.

For instance, the Campo de Dalías (Almería) SWRO illustrates the scale and technical profile of Spain’s large municipal/irrigation assets. Commissioned at ~97,200 m^3^/d (≈30 hm^3^/y) with double-pass RO for boron control and designed for 129,600 m^3^/d expansion, the plant integrates high-efficiency energy recovery and a 41 km distribution network serving agriculture and urban demand. Official project documentation (Acuamed) and subsequent engineering contracts confirm the nominal 30 hm^3^/y capacity and ongoing expansion toward 40 hm^3^/y, reflecting rising irrigation and urban needs in the Poniente Almeriense. The asset sits within a portfolio of Mediterranean-basin plants (e.g., Carboneras, Torrevieja, Alicante-Mutxamel) that collectively underwrite drought resilience in Southeastern Spain [20,21,22,23,24].

While non-conventional supply has increased reliability, Spain’s scientific community cautions that efficiency gains can be offset by rebound effects (e.g., irrigation expansion), and that ecological thresholds in rivers and aquifers require stronger protection during drought allocations. A 2025 perspective synthesizes risks to freshwater ecosystems under escalating scarcity, advocating ecosystem restoration, conservation agriculture, controls on illegal abstractions, and integrated economic-ecologic planning. The RBMPs (2022–2027) embed groundwater action and ecological-flow implementation, but effective outcomes depend on enforcement, precise metering, and digital monitoring of returns and losses in aging networks—areas singled out by macroeconomic and policy reviews.

By 2026, Spain’s trajectory is clear: (i) institutionalized reliance on desalination and reuse as permanent pillars of supply in stressed basins; (ii) alignment with EU reuse law and national RD 1085/2024 to standardize safety, monitoring, and transparency; and (iii) capital mobilization under the RBMPs to modernize sanitation, reduce non-revenue water, and upgrade irrigation infrastructure. The national narrative has shifted from emergency deployment to programmatic expansion: Catalonia, Andalusia, Murcia, the Balearics, and the Canaries are advancing new SWRO units, expansions, and hybridization with renewables to stabilize costs and curtail carbon intensity, while municipal utilities prepare urban reuse plans under the 2024 decree [3,8].

At the European scale, periodic EEA WEI+ updates will remain indispensable for distinguishing seasonal scarcity (often masked by annual national indicators) and for prioritizing basin-specific measures. For Spain, success by 2027 will hinge on three technical levers: (1) pretreatment-optimized SWRO (DAF-DMF vs. UF) tuned to intake variability and biofouling control; (2) low-carbon OPEX through demand response, high-efficiency energy recovery, and renewables integration; and (3) reuse mainstreaming with validated risk-management, transparent quality reporting, and targeted incentives for agricultural uptake under EU 2020/741 classes [21,22,23,24].

## 3. Brackish Water Reverse Osmosis: The Challenge Against Biofouling

Biofouling constitutes one of the most persistent and detrimental operational challenges faced by reverse osmosis (RO) facilities, and it has long been recognized as a critical vulnerability within membrane-based desalination systems. Its development exerts a profound influence on long-term process stability because biological deposits gradually increase hydraulic resistance both across the membrane surface and within the feed-spacer channels. This heightened resistance necessitates higher operating pressures to maintain production, ultimately elevating specific energy consumption and compromising overall system efficiency. As resistance builds, operators are compelled to conduct more frequent and intensive chemical cleanings to restore permeability. Such interventions, although indispensable, shorten membrane service life and contribute to escalating operational expenditures. Among all fouling mechanisms—particulate, organic, inorganic scaling—biofouling is considered uniquely problematic due to its capacity for self-sustaining growth and its resilience under fluctuating hydraulic and chemical conditions [18,19,20].

The difficulty in controlling biofouling stems from the biological structure of the deposits themselves. Biofilms consist of heterogeneous communities of microorganisms embedded in a protective extracellular matrix that confers structural stability and shields cells from disinfectants, oxidants, and mechanical shear. Once established, this matrix functions as a dynamic microenvironment where bacteria can adhere firmly, proliferate, and metabolically adapt to local stressors. RO membranes provide especially favorable conditions for biofilm formation: nutrients, even in trace concentrations, accumulate near the surface through concentration polarization, while the protective geometry of the feed channel reduces local shear forces. As a result, even low bacterial counts in the feed water can lead to significant biofilm development over time, particularly in systems sourcing water from surface or open-intake environments, where natural variability in organic matter and microbial load amplifies the risk.

To address this issue, a wide array of mitigation strategies has been implemented across modern RO systems. One major approach involves modifying membrane-surface chemistry to reduce the tendency of bacteria to adhere. Adjustments to surface hydrophilicity, charge density, and smoothness alter the physicochemical interactions between the membrane and microbial cells, making initial attachment less favorable. A smoother and more hydrophilic surface lowers the energy required for water–membrane interactions, while simultaneously increasing the energy barrier for microbial adhesion. In practice, this reduces the formation of initial conditioning films, which are precursors to mature biofilms. The introduction of stable, chemically resistant membrane formulations ensures that such surface modifications do not compromise mechanical or chemical robustness during operation or during cleaning cycles [6,7,8].

A second major engineering strategy involves optimizing the physical configuration of the feed-spacer mesh within the RO element. Increasing spacer thickness creates broader flow channels that enhance local turbulence and disrupt boundary-layer development. This increase in mixing reduces the accumulation of particulate matter and organic precursors that facilitate bacterial attachment. Furthermore, enhanced turbulence helps distribute shear forces more effectively across the membrane surface, making it more difficult for microorganisms to anchor and initiate biofilm formation. In addition, improved spacer geometries reduce the likelihood of feed-channel plugging, maintaining more uniform flow distribution and lowering the risk of localized stagnation zones—areas where biofouling can intensify rapidly.

The benefits of spacer optimization also extend to cleaning efficacy. Higher turbulence promotes more effective penetration of cleaning solutions during maintenance procedures, allowing chemical agents to detach biological material more efficiently. Consequently, systems designed with enhanced spacers often exhibit better long-term stability, lower cleaning frequency, and slower rates of performance decline compared with conventional designs. These engineering improvements work synergistically with membrane-surface modifications, creating a multilayered defense against biofouling that targets both the initial attachment phase and the subsequent growth of biological communities [12,13,14].

Overall, the integration of advanced membrane chemistries and optimized module geometries represents a significant evolution in RO technology. These developments address biofouling from both a material-science perspective—by modifying the membrane interface—and a hydraulic-engineering perspective—by controlling flow dynamics within the feed channel. When combined with appropriate pretreatment and operational strategies, such innovations substantially improve the resilience of RO systems, enabling more consistent performance under challenging source-water conditions and reducing the lifecycle costs associated with biofilm management [3,8].

Thanks to the thicker feed spacer more turbulence can be created along the feed channel, which makes it more difficult for bacteria and microorganism to get attached on the surface of the membrane. The same increase in turbulence favors the cleaning results [22,23,24].

## 4. Large Reverse Osmosis Module Diameter

A comprehensive evaluation carried out by the Large Diameter Membrane Manufacturers Consortium concluded that increasing the diameter of spiral-wound RO elements to 16 inches represents the most balanced engineering solution when considering the trade-offs between capital-cost reduction, the higher fabrication and installation costs associated with larger pressure vessels, and the operational risks inherent to scaling up membrane hardware. Their analysis identified this diameter as an optimal midpoint because it achieves meaningful economic benefits without introducing disproportionate mechanical or hydraulic complications. According to the consortium’s assessment—supported by an independent external engineering consultancy—the projected capital-expenditure savings resulting from increasing module diameter from 8 inches to 16 inches range from 7% to 11% for seawater RO installations and from 9% to 15% in brackish-water desalination systems. These reductions depend strongly on plant size, membrane array configuration, and the overall layout of the treatment system, but they consistently point toward improved cost-effectiveness at larger scales [3,8,12].

When the engineering team responsible for developing these enlarged modules began the design process, their strategy centered on a systematic scale-up from the widely deployed 8-inch commercial modules with long-established performance reliability. Because the active membrane sheet and fundamental separation properties remain the same in both formats, the principal design effort focused on addressing the structural and mechanical challenges introduced by the increased module diameter. In particular, substantial attention was dedicated to reinforcing the permeate collection tube to withstand higher mechanical loads and ensuring the robustness of the external fiberglass shell. Additionally, the end-cap configuration was redesigned to incorporate the advantages of the interlocking end-cap system, a feature intended to improve sealing integrity, reduce the risk of telescoping under high pressure, and simplify module alignment during installation. The resulting 16-inch elements achieve approximately 4.3 times the active surface area provided by a standard 8-inch module while maintaining the same rejection characteristics, enabling significantly higher permeate production per vessel without sacrificing separation performance.

Field validation of the new module size has been performed at the Bedok NEWater Plant in Singapore, operated by the national water utility. To evaluate performance under identical conditions, two parallel RO trains—one using conventional 8-inch modules and the other employing the scaled-up 16-inch elements—were operated simultaneously for a ten-month period. Both trains received the same pretreated feedwater, consisting of a blend of treated industrial and municipal effluent further conditioned by chloramination and ultrafiltration. Each train was configured with a 2:1 array of seven-module pressure vessels, and operational controls were adjusted to ensure equal average flux (17.3 LMH) and identical system recovery (75%). By maintaining matched operating conditions, the trial allowed for a direct comparison of the hydraulic behavior, stability, and long-term performance of both module sizes.

Throughout the monitoring period, both systems demonstrated highly consistent performance trends. As expected from the increased membrane area, the 16-inch train produced a permeate flow approximately 4.3 times higher than that of the 8-inch train when operated at equivalent hydraulic conditions. Normalized permeability data for both racks confirm this proportional enhancement. A temporary anomaly was observed between December 2007 and January 2008, when the 8-inch train exhibited a short-term increase in permeate flow. Upon investigation, plant operators determined that the 8-inch train had inadvertently undergone a backwashing effect because the 16-inch system remained in service while the smaller-diameter train was temporarily halted. This unintended interruption caused a removal of accumulated foulants and resulted in a transient rise in normalized production once the 8-inch train was restarted.

**Figure 6:**
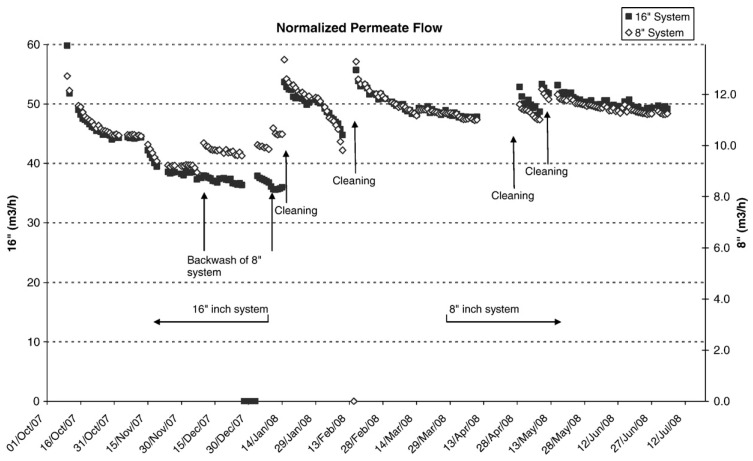
16-inch and 8-inch trains normalized permeate flow.

Notably, after scheduled caustic cleanings performed on both trains, the normalized flux values promptly returned to their initial levels, indicating that neither module size experienced irreversible performance deterioration during the trial. In terms of water quality, the two systems produced permeate with equivalent and stable salt rejection, confirming that increasing module diameter does not compromise desalination performance when structural considerations are properly addressed. Overall, the comparison demonstrates that 16-inch modules can reliably deliver substantially higher throughput while maintaining the same rejection behavior as their 8-inch counterparts, Figure 6, reinforcing their suitability for large-scale desalination applications where higher production per vessel translates directly into reduced capital costs and more compact system layouts [3,8,25].

## 5. Conclusions

The evaluation of reverse osmosis (RO) technologies presented in this study demonstrates clear progress in the performance, energy efficiency, and operational stability of desalination systems currently deployed in Spain. Recent membrane modules, including high-rejection and high-productivity seawater elements, show measurable improvements in solute removal and permeate output, enabling lower operating pressures and reduced specific energy consumption. Field data from full-scale installations confirm that modules such as FILMTEC^TM^ SW30XHR-400i and SW30ULE-400i can achieve enhanced boron rejection and sustained hydraulic performance under representative Mediterranean feedwater conditions.

System-level innovations, particularly Internally Staged Designs (ISD), provide a further performance advantage. By distributing membrane productivity along the pressure vessel, ISD configurations have demonstrated more uniform flux, lower fouling propensity, and increased permeate production without exceeding recommended operating limits. Operational records from Spanish seawater plants indicate stable permeability and salt rejection over extended periods, with reduced cleaning frequency compared with conventional arrangements.

The long-term evaluation of 16-inch membrane elements highlights their potential to reduce capital expenditure in large-scale facilities by increasing production per pressure vessel. Comparative trials show that these modules maintain equivalent rejection performance to 8-inch elements while delivering approximately fourfold higher permeate flow when operated under identical hydraulic conditions, confirming their suitability for high-capacity desalination plants.

Finally, the review of brackish-water RO systems underscores the continued importance of addressing biofouling, a dominant factor in long-term operational decline. Advances in membrane-surface chemistry and feed-spacer geometry contribute to mitigating microbial attachment and improving cleaning effectiveness, thereby supporting more stable operation in variable-quality inland sources.

Overall, the technological evidence compiled in this work reinforces the central role of RO in Spain’s desalination and water-reuse infrastructure. Improvements at the membrane, module, and system levels collectively enhance process efficiency, reliability, and cost-effectiveness, supporting the continued expansion and optimization of RO-based solutions in semi-arid and coastal regions.

Spain is currently undertaking a substantial and strategically coordinated effort to secure a resilient and sustainable water supply system. In parallel with the WATER program established in 2005, the recent approval of a national Royal Decree on water reuse represents a decisive regulatory step aimed at expanding water availability, optimizing the performance of existing distribution and treatment infrastructures, ensuring consistent supply quality, and minimizing associated environmental impacts. A considerable proportion of the measures outlined within this legislative framework inherently depend on the implementation of reverse osmosis (RO) technologies, whether for seawater and brackish water desalination or for advanced water reclamation applications.

Contemporary advancements in RO technology have been principally directed toward reducing the overall cost of treated water while maintaining or enhancing process efficiency [3,8].

The adoption of larger-diameter membrane modules has also emerged as a promising pathway for reducing the capital expenditure associated with large-scale RO facilities. A standardized 16-inch module diameter, recommended by the Large Diameter Membrane Manufacturers Consortium in 2004, was commercially available and undergoing long-term field evaluation. FILMTEC^TM^ 16-inch elements have exhibited robust and consistent performance under these trials, delivering approximately 4.3-fold higher permeate flow compared with conventional 8-inch modules when operated under identical hydraulic and thermodynamic conditions.

Building upon these technological advances, Spain’s broader water-management strategy is increasingly oriented toward integrating RO-based desalination and reuse systems into a flexible, climate-resilient supply portfolio. As hydrological variability intensifies and conventional sources become less predictable, the combination of high-efficiency membrane systems, energy-recovery technologies, and advanced pretreatment configurations offers a viable pathway to stabilize long-term water availability. The continued expansion of desalination capacity along coastal regions, together with the progressive incorporation of tertiary and quaternary treatment stages in wastewater-reclamation facilities, underscores the country’s commitment to diversifying water resources while minimizing the pressure on over-exploited aquifers and surface-water bodies. This integrated approach not only enhances supply security but also aligns with European regulatory directives aimed at promoting circular water use and reducing environmental impacts across the entire water cycle.

Equally important is the role of ongoing research, operational monitoring, and digital process optimization in ensuring that RO systems operate at maximum reliability and efficiency. Real-time performance assessment, data-driven maintenance strategies, and predictive modeling of fouling or scaling events are progressively being adopted to extend membrane lifespan and reduce energy consumption. These operational innovations, combined with continuous improvements in module design and system architecture, position reverse osmosis as a technologically mature and increasingly cost-competitive solution within Spain’s water-resource planning framework [3,8]. As these efforts advance, Spain is poised to consolidate its status as a European leader in the implementation of membrane-based desalination and water-reuse technologies, demonstrating how engineering innovation, regulatory modernization, and strategic investment can jointly underpin a sustainable and resilient national water supply system.

## Figures and Tables

**Table 1:** Water reuse and desalination priority actions included in the Spanish Organic Law (June 2005).

Area	Action	Project
Cuenca Hidrográfica del Sur Cuenca Hidrográfica del Segura Cuenca Hidrográfica del Júcar	Sea/brackish water desalination Water reuse Sea/brackish water desalination Water reuse Sea/brackish water desalination Water reuse	Campo de Dalías, Najar, Bajo Almanzora, Carboneras, Poniente Almeriense, Marbella, Costa del Sol Campo de Dalías, Almería, Costa del Sol Campo de Cartagena, Tajo-Segura, Canales del Taibilla (Murcia), Guadalentín, Murcia, La Pedrera, Pilar de la Horadada, Alacantí y Vega Baja, Campo de Cartagena, El Mojón Mar Menor, Murcia-Este, Hellín Marina Alta, Marina Baja, Canales del Taibilla (Alicante), Jávea Villajoyosa, Novelda y Monforte del Cid, Sueca, Albufera Sur, Vinalopó-Alacantí, Plana de Castellón, Monte Orgegia, Rincón de León, Elda-Petrer, Alcoià-Comtat, Ontinyent-Vall d’Albaida, Gandía, Xàbia, Oliva

**Table 2:** Operating conditions of a Seawater Reverse Osmosis plant operated with FILMTEC^TM^ SW30XHR-400i modules.

Feed Conductivity (µS/cm)	Feed pH	Feed Pressure (bar)	Feed Temperature (°C)	Recovery (%)	Boron Feed (ppm)	Boron Permeate (ppm)
48,000	7.4	58	18	48	4.84	0.55

## Data Availability

The raw data supporting the conclusions of this article will be made available by the authors on request.
